# The Emergence of Multidrug-Resistant *Helicobacter pylori* in Southeast Asia: A Systematic Review on the Trends and Intervention Strategies Using Antimicrobial Peptides

**DOI:** 10.3390/antibiotics10091061

**Published:** 2021-09-01

**Authors:** Asif Sukri, Bruno S. Lopes, Alfizah Hanafiah

**Affiliations:** 1 Integrative Pharmacogenomics Institute (iPROMISE), Universiti Teknologi MARA (UiTM), Selangor Branch, Puncak Alam Campus, Puncak Alam 42300, Malaysia; mohdasifsukri@gmail.com; 2 Department of Medical Microbiology, School of Medicine, Medical Sciences and Nutrition, University of Aberdeen, Aberdeen AB25 2ZD, UK; brunoldlopez@gmail.com; 3 Department of Medical Microbiology and Immunology, Faculty of Medicine, Universiti Kebangsaan Malaysia, Kuala Lumpur 56000, Malaysia

**Keywords:** *Helicobacter pylori*, low and middle-income countries, antimicrobial resistance, antimicrobial peptides, multidrug resistance, clarithromycin, metronidazole

## Abstract

The emergence of multidrug-resistant *H. pylori* poses a public healthcare threat, particularly in low- and middle-income countries. Recently, the World Health Organization has classified clarithromycin-resistant *H. pylori* as high priority in the research and discovery of novel antibiotics. This study was aimed to systematically review the prevalence of primary antibiotic resistance in *H. pylori* in Southeast Asian countries (SEAC) and to review current studies of antimicrobial peptides against *H. pylori*. We systematically searched through electronic databases of studies conducted on antimicrobial resistance of *H. pylori* in SEA countries. Furthermore, we searched articles that conducted studies on antimicrobial peptides, naturally occurring host’s defense molecules, against *H. pylori*. After a series of screening processes, 15 studies were included in our systematic review. Our analysis revealed that primary resistance of *H. pylori* to metronidazole, clarithromycin, and levofloxacin were high in SEAC, although the primary resistance to amoxicillin and tetracycline remains low. Multidrug-resistant *H. pylori* are emerging in SE Asian countries. The antimicrobial peptides show promising antibacterial and antibiofilm activity against drug-resistant *H. pylori*. The research and discovery of antimicrobial peptides against *H. pylori* in SEAC will help in limiting the spread of antimicrobial resistance of *H. pylori*.

## 1. Introduction

*Helicobacter pylori* is one of the most common infectious disease agents worldwide, with more than 50% of the world’s population being infected with this pathogen, mostly in developing countries [1]. Transmission of *H. pylori* is still uncertain, but several studies have shown that close contact of a mother with a child during childhood is the main transmission route, apart from drinking contaminated water [2]. Since its discovery by Warren and Marshall almost three decades ago, it is now established that this bacterium orchestrates gastric carcinogenesis by producing multiple virulence factors that lead to peptic ulcer and gastric cancer [3]. Gastric cancer is still one of the most common cancers globally, with more than 1 million new cases reported yearly, leading to 768,793 deaths in 2020 alone [4]. Furthermore, gastric cancer patients are mainly detected at the advanced stage of cancer, where cancer prognosis is worse than that of detection at an early stage [5]. A high economic and mortality burden is associated with gastric cancer, with most cases occurring in developing countries where medical resources for early screening and patient management are limited [4]. Therefore, early screening of gastric cancer for detecting precancerous lesions for better prognosis and eradicating *H. pylori* are two essential strategies to prevent gastric cancer development.

Treatment of *H. pylori* includes administering proton pump inhibitors (PPI) coupled with multiple antibiotics through several treatment regimens. In the first-line triple therapy, PPI is administered with amoxicillin and clarithromycin. However, in areas where the clarithromycin resistance rate is higher than 15%, triple therapy containing metronidazole or bismuth-containing quadruple therapy is recommended [6]. Nevertheless, the increasing rate of multiple-drug-resistant *H. pylori* has implications for the eradication of bacteria to prevent gastric diseases, including gastric cancer and ulcers [7]. The World Health Organization (WHO) has declared clarithromycin-resistant *H. pylori* a high priority in the research and development of novel antimicrobial discovery [8]. As such, an alarming concern of the increasing rate of antibiotic resistance presents urgency for the discovery of novel or alternative therapies for *H. pylori*. SEAC are home to more than 650 million people with diverse ethnicities and cultures. Most of these countries are mainly low-and middle-income countries, where medical resources are limited [9]. However, it is also home to diverse natural flora and fauna, with more than 20% of global plant and animal species, and where four out of twenty-five global biodiversity hotspots are found [10]. Antimicrobial peptides (AMP) are a small class of peptides that are part of an organism’s innate immunity with an inhibitory effect against pathogens such as the inhibition of nucleotide and protein synthesis of pathogens, formation of the pore at the cell membrane, and the host’s immunomodulation [11]. AMPs are good candidates for alternative therapies for the treatment of antibiotic-resistant infections and are broad-spectrum antimicrobial agents to which most bacteria evolution is slow. AMP has been isolated from multiple flora and fauna such as amphibians, fish, and plants [12]. Given that SEA has one of the world’s greatest biodiversity hotspots, this region can be one in which AMP research and discovery can be conducted rapidly. The objective of this review was to systematically study antibiotic resistant in *H. pylori* in SEA and to summarize our current understanding of AMP and its role as an alternative therapeutic option for *H. pylori* infections.

## 2. Results

**Figure 1:**
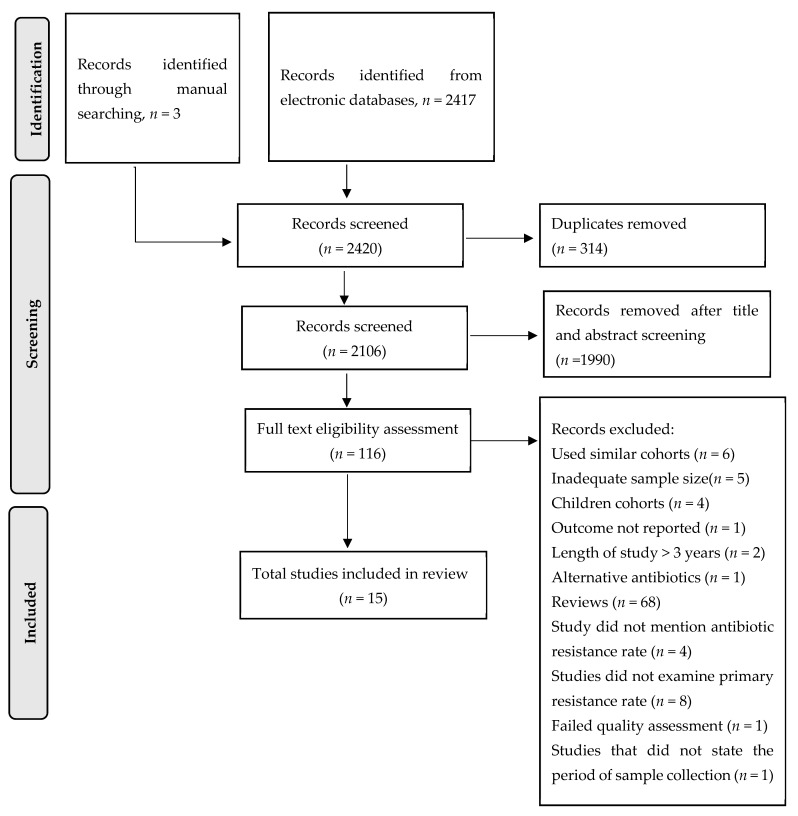
Flow chart of study selection. Exclusion criteria were: (1) Studies that measured the prevalence of alternative antibiotics for *H. pylori* treatment, (2) studies that reported prevalence results using < 50 isolates for culture method or <50 *H. pylori* positive gastric biopsies for nucleic acid-based method, (3) studies that only reported percentage of resistance rates with no description of the number of isolates used, (4) studies with no time frame of sample collection, (5) studies that clustered prevalence rates data more than three years, (6) studies that failed to state the percentage of primary antibiotic resistance in *H. pylori* isolates examined, and (7) studies that only examined the prevalence of resistance rate of secondary isolates.

The systematic search through electronic databases resulted in 2417 articles, and an additional three articles were found by checking references of a previous systematic review. After title and abstract screening, 116 articles underwent full-text assessment (Figure 1). By applying inclusion and exclusion criteria, only 16 articles from 7 out of 10 SEA countries met the eligibility criteria for inclusion in this systematic review. After quality assessment, one article was excluded due to low quality (quality score = 3), and the final number of articles included in this systematic review was 15. The articles from countries that were included in this systematic review were as follows: Cambodia (*n* = 1), Indonesia (*n* = 1), Laos (*n* = 1), Malaysia (*n* = 3), Singapore (*n* = 1), Thailand (*n* = 5), and Vietnam (*n* = 3) and were published between 2011 to 2020. The patients recruited in the studies were diagnosed with symptoms of dyspepsia. The mean patient age in the included studies ranged from 38 to 55 years old. The methods employed to diagnose *H. pylori* infection in the included studies were bacterial culture, rapid urease test, molecular technique of polymerase chain reaction (PCR), and histopathological examination. For antibiotic resistance testing, a molecular technique and a culture-based method, namely the E-test and the agar dilution assay, were used (Table 1).

### 2.1. Summary of Primary Antibiotic Resistance Rate in H. pylori

#### 2.1.1. Clarithromycin Resistance

From our systematic search, 15 studies from SEA countries examined primary clarithromycin resistance in *H. pylori* (Table 2). In Thailand, clarithromycin resistance ranged from as low as 5.6% to as high as 76.2% for four studies conducted from 2013 to 2017. Of the four studies, the trend of clarithromycin resistance in Thailand was 5.6% in 2013 [15], 76.2% in 2014 to 2015 [17], 2% in 2016 [14], and 12.9% in 2017 [13]. The variability of clarithromycin resistance in *H. pylori* isolated in Thailand can be attributed to locations where the sampling was conducted and the type of diagnostic test used to assess antibiotic resistance. The prevalence of clarithromycin resistance was as high as 76.2% from the samples collected in the Northeast region of the country and molecular detection was used instead of culture-based detection [17], while the prevalence was less than 13% from the samples collected from the Mountain people [15], namely Karen, Thai, and Hmong people, the Pathumthani region [13], and the Chiangrai province [14] using culture-based diagnosis. In Cambodia, one study conducted in the country revealed primary clarithromycin resistance of *H. pylori* as 25.5% in 2015 [18] while in Indonesia, the clarithromycin resistance rate was 9.1% from 2012 to 2015 [19]. In Laos, the clarithromycin resistance rate was 12.6% from 2010 to 2012 [20]. Three studies examined primary clarithromycin resistance in *H. pylori* in Malaysia, in which the resistance rate showed an increasing trend from 2004 to 2015. From 2004 to 2009, the clarithromycin resistance rate was <2.1% [22,23]. However, the resistance rate increased to 12.2% from 2014 to 2015 [21]. Meanwhile, in Singapore, a study published in 2015 revealed the primary clarithromycin resistance was 17.9% [24]. In Vietnam, the primary resistance rate of clarithromycin increased from 33% in 2008 [26] to 34.2% from 2012 to 2014 [27] and 66.1% from 2014 to 2016 [25].

#### 2.1.2. Metronidazole Resistance

Fifteen studies examined the primary resistance rate of metronidazole resistance in *H. pylori*, of which all studies found the resistance rate was high (>15% resistance rate). In Thailand, the metronidazole resistance rate showed an increasing trend from 30% between 2008 to 2010 [16] to 71.8% in 2013 [18] and 62.8% in 2017 [13]. A similar trend was also observed in Malaysia, in which the metronidazole resistance rate ranged from 36.9% [23] to 75.5% [22] from 2004 to 2009 and 56.1% between 2014 to 2015 [21]. In Vietnam, the trend of the metronidazole resistance rate was 69.9% in 2008 [26] and increased to 75.3% from 2012 to 2014 [27]. In Singapore, the metronidazole resistance rate observed was 48.1% from 2011 to 2014 [24]. The resistance rate was alarmingly high in Laos, in which a recent observation in 2015 revealed the rate was 96.4% [18]. Meanwhile, in Indonesia, the resistance rate of clarithromycin resistance in *H. pylori* was 46.8% from 2012 to 2015 [19].

#### 2.1.3. Levofloxacin Resistance

The primary levofloxacin resistance rate was observed to increase from year to year in Thailand, in which the resistance rate increased from 19.4% [15] in 2013 and 22% in 2016 [14]. Meanwhile, high resistance rate of levofloxacin was observed in Cambodia [18] and Indonesia [19] (67.3% and 31.2%, respectively). In Malaysia, the primary resistance rate of levofloxacin was high at 17.1% from 2014 to 2015 [21]. The trend of levofloxacin resistance rate increased in Vietnam from 18.4% in 2008 [26], 35.6% from 2012 to 2014 [27] to 38.1% from 2014 to 2016 [25].

#### 2.1.4. Amoxicillin Resistance

Our analysis revealed that the primary amoxicillin resistance rate of *H. pylori* in SEAC is still low. No *H. pylori* isolate was reported to be resistant to amoxicillin in Malaysia [21,22,23] while in Thailand, the resistance rate of *H. pylori* to amoxicillin was reported to be 0.8% in a study conducted from samples collected in 2013 [15] while no amoxicillin resistance isolate was observed in another study [14]. In Cambodia and Indonesia, rates of amoxicillin resistant *H. pylori* were 9.1% [18] and 5.2% [19], respectively. In Singapore, the resistance rate was 4.7% from 2011 to 2014 [24] while in Vietnam, the resistance rate was not observed in the study conducted from 2012 to 2014 [27].

#### 2.1.5. Tetracycline Resistance

Similar to the amoxicillin resistance rate, the tetracycline resistance rate in most SEACs is low. No reports of tetracycline-resistant *H. pylori* were observed in Thailand and Malaysia, while in Indonesia, the resistance rate was 2.8% [19]. However, in Vietnam, the tetracycline resistance rate was reported to be 5.8% in 2008 [26].

#### 2.1.6. Multidrug Resistance Rate of *H. pylori*

The multidrug resistance rate of *H. pylori* reported in SEA countries varied across all countries examined. In Malaysia, the reported multidrug resistance was still low, but than increasing resistance rate was observed. Of the two studies that reported a multidrug resistance rate in Malaysia, the resistance rates were 2.1% from 2004 to 2007 [23] but increased to 7.4% from 2014 to 2015 [21]. This finding suggests that there is an increase in multidrug-resistant *H. pylori* in Malaysia. Similarly, Singapore, the neighboring country of Malaysia, had a multidrug resistance rate of 7.5% between 2011 to 2014 [24]. Of the two studies that reported multidrug resistance rate in Thailand, one study found that the rate was 4% in 2016 [14] and 21.8% in 2013 [15]. Contrary to Malaysia, Thailand, and Singapore, the multidrug resistance rates of *H. pylori* in Vietnam and Cambodia were high. The rate of multidrug-resistant *H. pylori* in Vietnam increased from 24.3% in 2008 [26] to 30.7% from 2014 to 2016 [25]. Meanwhile, the multidrug resistance rate of *H. pylori* strains in Cambodia was extremely high at 76.4% [18], of which 40% were resistant to levofloxacin and metronidazole, 7.3% were resistant to clarithromycin and metronidazole, 9.1% were resistant to metronidazole, levofloxacin, and amoxicillin, and 18.2% were resistant to clarithromycin, levofloxacin, and metronidazole.

### 2.2. A Review on Intervention Strategy Using AMP

Given the high resistance rates of *H. pylori* to critically important antibiotics in this region, we searched the database on intervention strategies of *H. pylori* treatment using AMP. AMPs are small peptides that have been demonstrated to possess broad-spectrum activity against bacteria. They are usually isolated from natural sources such as the skin of amphibians and venoms. The SEACs are a region with high biological diversity and lush tropical forests where the sources for discovery and research on novel antimicrobial drugs against *H. pylori* can be further explored. As such, we aimed to summarize the studies conducted on AMPs against *H. pylori* for understanding the current progress in research. Table 3 summarizes the studies conducted on AMPs against *H. pylori*. We found that all the studies conducted demonstrated antibacterial activity of AMPs against *H. pylori* and all studies employed synthetically synthesized AMPs (Table 3). Five AMPs, namely cathelicidin [28], bicarinalin [29], odorranain-HP [30], tilapia Piscidin 4 [31], and pleurain-A [32], were initially identified from natural sources and then synthesized synthetically in the laboratory. Notably, some AMPs, including cathelicidin-like AMP [33], bicarinalin [29], fusion human neutrophil peptide 1 [34], and epinecidin 1 [35], showed bactericidal activity against drug-resistant *H. pylori*. The mechanism of bactericidal and bacteriostatic AMPs includes the perturbation, permeabilization, and rupture of the bacterial cell membrane (Table 3). In addition, cathelicidin from humans and mice exhibited antibiofilm activity against *H. pylori* and protected the animal model from inflammation induced by *H. pylori* [28]. A study conducted by Jiang et al. [33] found that a cathelicidin-like peptide, namely Cbf-K_16_, reduced intercellular and intracellular activity of *H. pylori* and decreased *H. pylori* colonization in animal model. Furthermore, helix-coil conformation transitional antimicrobial polypeptides demonstrated bactericidal activity against *H. pylori* at low pH, suggesting its potential for use in human. Besides that, the peptide demonstrated low toxicity in animal study and an examined stomach, suggesting that the peptide is worthy to be evaluated in clinical trials [36].

## 3. Discussion

This systematic review consists of the studies conducted in the SEA region in countries with a diverse ethnic background, culture, and socioeconomic status. Our systematic review revealed that primary resistance of metronidazole in the SEA region is high with a more than 15% threshold while the primary resistance to clarithromycin varies according to the country, i.e., high in Singapore, Cambodia, and Vietnam, medium in Malaysia, and still low in Indonesia. In countries such as Thailand and Laos, resistance rates to clarithromycin are increasing and would exceed the threshold of 15% if the intervention strategy is not implemented. From the data published, resistance rates to levofloxacin also appeared to be high (>15%) in all the SEA countries, with the rate observed to be as high as >60% in Cambodia. Meanwhile, the resistance rates of *H. pylori* to amoxicillin and tetracycline are still low, with Malaysia reporting no cases of amoxicillin- and tetracycline-resistant *H. pylori* (Table 2). However, the amoxicillin- and tetracycline-resistant *H. pylori* have been reported in Indonesia and Cambodia, suggesting the emergence of *H. pylori* resistant to both antibiotics in these countries. Our findings are consistent with previous systematic reviews conducted by Savoldi et al. [7], which found that the resistance rates of *H. pylori* to metronidazole, clarithromycin, and levofloxacin are high in the SEA region. A similar systematic review also revealed high resistance rates of metronidazole in other parts of the world, including the Americas region, the European region, the Eastern Mediterranean region, and the Western Pacific region. Kuo et al. [42] also found an increasing rate of clarithromycin and metronidazole resistance in *H. pylori* in the Asian Pacific region. The rate of metronidazole resistance in SEA countries was observed to be very high (26–96.4%) in all SEA countries included in this systematic review. Metronidazole is used for treatments including parasitic infections, oral infections, gynecological infections, bone and joint infections, septicemia, and endocarditis [43], and is one of the drugs recommended as a first-line therapy in the SEA *H. pylori* management consensus [44]. The high resistance rate of metronidazole in this region could not be ascertained, although excessive use of the antibiotics and non-compliance among patients in treatment regimens are factors associated with the rising antibiotic resistance rate [45]. Trends of clarithromycin resistance also show a sharp increase from year to year in Malaysia, with <2.1% from 2004–2009 [22,23] to as high as 12.2% in 2014–2015 [21]. Vietnam also showed a sharp increase of clarithromycin resistance from 33% in 2008 [26] to 72.6% from 2014–2016 [25] while the trend in Thailand fluctuated from year to year. The trend of antimicrobial resistance rates in other countries could not be assessed due to a lack of studies conducted on antimicrobial resistance in *H. pylori*. This factor could be due to the difficulty of culturing *H. pylori* in the laboratory and the high cost of performing nucleic acid-based technique in the SEA region, which mostly consist of low- and middle-income countries. The Maastricht V/Florence Consensus Report suggests that primary treatment consisting of clarithromycin should be abandoned if the resistance rate to clarithromycin in the region exceeds 15%, and quadruple therapy should be administered instead for successful eradication of *H. pylori* [6]. In the SEA region, the ASEAN Bangkok Consensus Report in 2016 recommends triple therapy consisting of clarithromycin as a first-line regimen for 14 days, provided that the prevalence of clarithromycin resistance in the area is less than 15%. However, the triple therapy consisting of clarithromycin should be replaced with quadruple therapy when clarithromycin resistance rate exceeds 15% [44]. A systematic review and meta-analysis on the impact of eradication regimens in the areas with high clarithromycin and metronidazole resistance reveals that sequential therapy is more effective in eradicating *H. pylori* than that of prolonged 14-day triple therapy, while there was no difference observed between sequential and 14-day triple therapy in the areas with high metronidazole resistance [46]. While prevalence data of clarithromycin is known in some SEA countries, the prevalence in other countries is unknown. This will complicate the treatment options for *H. pylori* in these regions. Findings from our systematic review reveals that clarithromycin resistance in SEA countries is increasing. Taken together, these results suggest that the primary treatment consisting of quadruple therapy or sequential therapy must be administered to *H. pylori*-infected patients in SEA countries with high prevalence of clarithromycin resistance, provided clinical data of patients are available prior to the administration. Besides clarithromycin resistance in *H. pylori*, clinical factors that contribute to first-line treatment failure also include the patient’s non-compliance during therapy period, the patient’s age (eradication was more successful in older patients), and the type of ulcer, in which eradication was significantly more successful in duodenal ulcers compared to gastric ulcers, which should be taken into consideration before switching to quadruple treatment [47].

In our systematic review, we also found an increasing rate of multidrug-resistant *H. pylori* in the SEA countries examined. In Cambodia, the resistance rate of multidrug-resistant *H. pylori* was alarmingly high at 76.4% [18], suggesting the urgency of an intervention strategy in this country. The multidrug-resistance of *H. pylori* in Vietnam was also high and increased from year to year [25,26], while the trend was also observed in Malaysia, where the prevalence of multidrug-resistant *H. pylori* increased from year to year although the resistance rate was still low [21]. Similar to Malaysia, the multidrug-resistant *H. pylori* observed in Singapore [24] was also low while the rate in Thailand fluctuated from year to year [14,15]. Some studies in our systematic review did not report the prevalence of multidrug-resistant *H. pylori*; hence we were unable to conclude. However, we hypothesize that the resistance rates of *H. pylori* to multiple drugs are increasing in this region based on the available data from the countries that had conducted study on the surveillance rate of multidrug-resistant *H. pylori* in their respective countries. As such, we suggest for future studies to include the prevalence of multidrug-resistant *H. pylori* when reporting the surveillance of antimicrobial resistance in *H. pylori*.

As the emergence of antimicrobial resistance in *H. pylori* to important antibiotics keeps increasing, WHO has classified clarithromycin-resistant *H. pylori* as a high-priority pathogen in the research and discovery of novel antibiotics [8]. AMPs have emerged as a new therapeutic option for the treatment of bacterial infection as they possess antimicrobial spectrum characteristic against bacteria. Most antimicrobial peptides have been isolated from natural sources, which suggest that the SEA region can be a hotspot for the research and discovery of new AMPs [11]. As such, we narratively summarized the research and discovery of AMPs against *H. pylori*. While the results of AMPs against drug-resistant *H. pylori* are promising and effective, we found that the study of AMPs against *H. pylori* are still lacking globally and there was no study conducted on AMPs against *H. pylori* in SEAC. Furthermore, our review also found that the clinical trial of AMP against *H. pylori* has yet to be conducted and all studies were either limited to animal or cell culture investigations. The challenges in the application of antimicrobial peptides for treatment include the cost-effectiveness of the novel drug to the economic health and the concern of toxicity associated with AMP [12]. As the SEA region consists of mostly low- and middle-income countries, the greatest challenge is the cost of AMP synthesis and whether it is economically cost-effective for the general population in this region. However, the research on AMP in the SEA region is worth being explored further as this region is rich in tropical biodiversity, a source for new antimicrobial peptide discovery.

The strength of our systematic review on the primary antibiotic resistance of *H. pylori* includes the examination of the trend of resistance rate in the SEA region that consists mostly of low- and middle-income countries. Our systematic review also informs policymakers regarding the alarming rate of *H. pylori* resistance to essential antibiotics such as clarithromycin and metronidazole in this region, suggesting an urgency for an intervention program. Furthermore, we presented a brief narrative review on the progress of AMP against *H. pylori* research worldwide with paucity of the research in the SEA region where the biodiversity hotspot exists. However, our systematic review is constrained by a lack of studies conducted on the surveillance of antimicrobial resistance in *H. pylori* in the SEA region, suggesting the need for future surveillance programs in different countries. In conclusion, the prevalence of clarithromycin- and metronidazole-resistant *H. pylori* is high in most SEA countries and will continue to rise in the future if intervention strategies are not adopted. While the resistance rates to amoxicillin and tetracycline are still low, there is a need for the development of an intervention strategy by policymakers to guide clinicians and respective bodies in this region to encourage research and discovery of new antimicrobial agents for the treatment of *H. pylori*. More research collaboration among SEA countries should be conducted for this matter, with harmonization of data and an understanding of any successful intervention strategies in specific countries.

## 4. Methods

### 4.1. Search Strategy and Study Selection

In the systematic review of antibiotic resistance in SEAC, we searched the potential research articles through electronic databases, namely PubMed, Scopus, Medline, and Science Direct, from 1 January 1990 to 1 May 2021. The electronic search was conducted on 6 May 2021. Additionally, we manually searched the articles by checking references from review and research articles relevant to our research questions. Details on specific keywords used for our search strategy using electronic databases are as follows:

((“Helicobacter pylori”) AND (“antibiotic” OR “antimicrobial” OR “anti-microbial” OR “antibacterial” OR “anti-bacterial” OR “drug”) AND (“resistance” OR “resistant*”) AND (“Malaysia” OR “Singapore” OR “Thailand” OR “Indonesia” OR “Brunei” OR “Myanmar” OR “Vietnam” OR “Timor Leste” OR “Laos” OR “Cambodia” OR “Philippines”)).

This systematic review was prepared according to Preferred Reporting Items for Systematic Reviews and Meta-Analysis (PRISMA) guidelines [48]. We included studies performed on populations in SEAC as defined by the United Nations [49]. These countries include Malaysia, Thailand, Singapore, Brunei Darussalam, Indonesia, Myanmar, Cambodia, Laos, Vietnam, and Philippines. Studies performed on populations outside SEA countries or studies without a clear population description were excluded from this systematic review. We also excluded studies conducted on children.

All study types were included in our search. However, we restricted our search to publications in English only. Abstracts without full text, all types of reviews, letters to the editors, and conference posters or presentations were excluded from our study. We included studies that measured the prevalence of primary antibiotic resistance for the following antibiotics: Metronidazole, clarithromycin, amoxicillin, tetracycline, and levofloxacin. Exclusion criteria were: (1) Studies that measured the prevalence of alternative antibiotics for *H. pylori* treatment; (2) studies that reported prevalence results using less than 50 isolates for culture method or less than 50 *H. pylori* positive gastric biopsies for nucleic acid-based method; (3) studies that only reported the percentage of resistance rates with no description of the number of isolates used; (4) studies with no time frame of sample collection; (5) studies that clustered prevalence rates data more than three years; (6) studies that failed to state the percentage of primary antibiotic resistance in *H. pylori* isolates examined; and (7) studies that only examined the prevalence of resistance rate of secondary isolates. If duplicate studies reported prevalence data using similar *H. pylori* cohorts, only the first study conducted on antibiotic resistance prevalence was chosen to be included in our systematic review.

### 4.2. Definitions of Diagnostic Tools and Antibiotic Resistance

Patients were defined as *H. pylori*-infected if they tested positive using one of the following diagnostic tools: Urea breath test, histopathological examination, culture-based test, rapid urease test of biopsies, and nucleic acid-based test. Antibiotic susceptibility tests include culture-based methods (E-test and the agar dilution method), or the nucleic acid-based test performed on *H. pylori* isolates or directly from gastric biopsies. Primary resistance was defined as *H. pylori* resistance to any antibiotics prior to administration of treatment regimens. Multidrug resistance in *H. pylori* is defined as *H. pylori* resistant to two or more antimicrobial agents. The threshold of high antibiotic resistance is a rate of more than 15% [6].

### 4.3. Data Extraction

Two independent reviewers independently screened the eligibility of articles from our search strategy through a two-step process. First, the articles were screened for title and abstracts for relevancy to our review. The full texts were retrieved and evaluated for the articles that passed the title and abstract screening process. Disagreement on inclusion or exclusion of the articles into the review was resolved through discussion and consensus. Data extracted from included studies were authors, country, year, period of sample collection, study design, patients’ clinical information (sample size, age, sex, histological findings, diseases, and *H. pylori* infection diagnostic tool used), and *H. pylori* samples (sample size, number of resistances, type of tested samples, the method to detect antibiotic resistance, and breakpoint system used). All data were extracted and sorted into Microsoft Office Excel 2016.

### 4.4. Quality Assessment of Studies

The quality of included studies was assessed as previously described [50], in which the score of study quality ranged from 0 to 8. A study with a score > 5 was considered as high quality while a study with a score that ranged 4–5 was considered as medium quality. Finally, a study with a score less than 4 was considered as low quality and would be excluded from this systematic review. Two reviewers independently scored each included article and any inconsistency on score result was resolved through discussion for final consensus.

### 4.5. A Narrative Review on AMP Studies in H. pylori

We searched through electronic literature databases on the studies using AMP in *H. pylori* treatment and summarized the findings.

## Figures and Tables

**Table 1:** List of studies included in the systematic review.

Author	Year Published	Patient’s Age (Mean in Years)	Country	No. ofPatients	HP Diagnostic Test	Patients’ Diseases	Antibiotic Resistance Method	Ref.
Auttajaroon et al.	2019	54.5	Thailand	93	RUT and culture	Functional dyspepsia	E-test	[13]
Vilaichone et al.	2017	55.9	Thailand	148	RUT and culture	Dyspepsia	E-test	[14]
Vilaichone et al.	2016	46.7	Thailand	291	RUT and culture	Dyspepsia	E-test	[15]
Vilaichone et al.	2011	49.1	Thailand	412	Culture and CLO test	Dyspepsia	E-test	[16]
Tongtawee et al.	2015	45.2	Thailand	300	HPE and RUT	Dyspepsia	Molecular technique ^a^	[17]
Tuan et al.	2019	45.3	Cambodia	206	Culture	NA	Agar dilution assay	[18]
Miftahussurur et al.	2016	49.6 (male) and 48.9 (female)	Indonesia	849	Culture and HPE	Dyspepsia	E-test	[19]
Vannarath et al.	2016	46	Laos	329	CLO and HPE	Dyspepsia	Molecular technique ^a^	[20]
Hanafiah et al.	2019	52.41	Malaysia	288	Culture, HPE and RUT	Chronic dyspepsia	E-test	[21]
Goh et al.	2011	50.5	Malaysia	90	Culture	NA	E-test	[22]
Ahmad et al.	2011	NA	Malaysia	777	Culture, RUT and HPE	Gastritis, peptic ulcer and gastric cancer	E-test	[23]
Ang et al.	2015	48	Singapore	462	UBT, RUT and HPE	NA	Agar dilution method	[24]
Dang et al.	2020	38.3	Vietnam	153	Culture and RUT	Chronic gastritis	E-test	[25]
Binh et al.	2013	47.3 (male) and 42.3 (female)	Vietnam	NA	Culture	Dyspepsia	E-test	[26]
Phan et al.	2015	44.1	Vietnam	92	Culture	Dyspepsia	E-test	[27]

^a^ Point mutation of A2143/2142CG in 23S rRNA to detect clarithromycin resistance; Abbreviations HP: *H. pylori*; RUT: Rapid urease test; HPE: Histopathological examination; CLO: *Campylobacter*-like organism; E-test: Epsilometer test; UBT: Urea breath test; CLA: Clarithromycin; MET: Metronidazole; LEVO: Levofloxacin; AMX: Amoxicillin; TET: Tetracycline; NA: Not available.

**Table 2:** Primary antibiotic resistance rates of *H. pylori*.

Author	Period of Sample Collection	Country	No. of HP Isolates	Primary Antibiotic Resistance Rate, %					MDR Rate, % (No. of Isolates)	Reference
				CLA	MET	LEVO	AMX	TET		
Auttajaroon et al.	2017	Thailand	93 ^a^	12.9	62.8	-	-	-	-	[13]
Vilaichone et al.	2016	Thailand	50	2	26	22	0	0	4% (2/50)	[14]
Vilaichone et al.	2013	Thailand	124	5.6	71.8	19.4	0.8	0	21.8% (27/124)	[15]
Vilaichone et al.	2008–2010	Thailand	100	-	30	-	-	-	-	[16]
Tongtawee et al.	2014–2015	Thailand	300 ^b,^*	76.2 ^c^	-	-	-	-	-	[17]
Tuan et al.	2015	Cambodia	55	25.5	96.4	67.3	9.1	0	76.4% (42/55)	[18]
Miftahussurur et al.	2012–2015	Indonesia	77	9.1	46.8	31.2	5.2	2.6	-	[19]
Vannarath et al.	2010–2012	Laos	119 ^b,^**	12.6	-	-	-	-	-	[20]
Hanafiah et al.	2014–2015	Malaysia	59 ^d^	12.2	56.1	17.1	0	0	7.4% (2/27)	[21]
Goh et al.	2009	Malaysia	90	0	75.5	0	0	-	-	[22]
Ahmad et al.	2004–2007	Malaysia	187	2.1	36.9	-	0	0	2.1% (4/187)	[23]
Ang et al.	2011–2014	Singapore	106	17.9	48.1	-	4.7	-	7.5% (8/106)	[24]
Dang et al.	2014–2016	Vietnam	153 ^d^	66.1	-	38.1	-	-	30.7% ^e^ (47/153)	[25]
Binh et al.	2008	Vietnam	103	33	69.9	18.4	0	5.8	24.3% (25/103)	[26]
Phan et al.	2012–2014	Vietnam	92 ^d^	34.2	75.3	35.6	0	-	50.7% (37/73) for primary resistance and 78.9% (15/19) for secondary resistance	[27]

^a^ Metronidazole resistance was performed in 43 isolates while clarithromycin resistance was performed in 70 isolates; ^b^ indicates antibiotic susceptibility test was conducted directly on gastric biopsies using molecular technique without isolation of the bacteria; ^c^ 76.2% was derived from the number of isolates that harbored both mutation and wildtype variants in 23S rRNA sequence; ^d^ Total number of isolates were derived from primary and secondary isolates. The primary resistance rates were recalculated using total primary isolates as denominator; ^e^ Multidrug resistance from both primary and secondary isolates; * DNA was extracted from all 300 gastric biopsies for molecular detection of antibiotic resistance, ** DNA was extracted from 119/329 patients for molecular detection of antibiotic resistance. Abbreviations: HP: *H. pylori*; CLA: Clarithromycin; MET: Metronidazole; LEVO: Levofloxacin; AMX: Amoxicillin; TET: Tetracycline; -: Not available.

**Table 3:** Summary of studies conducted on antimicrobial peptides against *H. pylori*.

Author	Year	Name of AMP	Source	Finding on Antibacterial Activity against *H. pylori*	Reference
Zhang et al.	2016	Cathelicidin	Mouse and human	Bactericidal activity against clarithromycin-resistant *H. pylori*; anti-biofilm activity against *H. pylori* SS1 strain; protected mouse from *H. pylori* orchestrated inflammation and reduced *H. pylori* colonization	[28]
Guzman et al.	2018	Bicarinalin	Synthetically synthesized in lab (anti venom *Tetramorium bicarinatum*)	Perturbation of membrane permeability against drug-resistant *H. pylori*	[29]
Chen et al.	2007	Odorranain-HP	Synthetically synthesized in lab (Diskless odorous frog, *Odorrana graham*)	Showed antimicrobial activity against *H. pylori* (MIC of 20 µg/mL)	[30]
Narayana et al.	2015	Tilapia Piscidin 4 (TP4)	Synthetically synthesized in lab (Nile tilapia, *Oreochromis niloticus*)	Demonstrated potential lytic activity against *H. pylori* surface membrane; disrupted the bacterial cell membrane	[31]
Wang et al.	2007	Pleurain-A	Synthetically synthesized in lab (Yunnan frog, *Rana pleuraden*)	Inhibited growth of *H. pylori* in vitro (30 µg/mL)	[32]
Jiang et al.	2020	Cbf-K_16_(cathelicidin-like AMP)	Synthetically synthesized	Demonstrated bactericidal activity against clarithromycin- and amoxicillin-resistant *H. pylori*; reduced intercellular and intracellular drug-resistant *H. pylori* in cell culture; showed increased membrane permeation in drug-resistant *H. pylori*	[33]
Zhang et al.	2018	Fusion human neutrophil peptide 1	Expression system in yeast	Eradication of wild type and drug-resistant *H. pylori* in animal model	[34]
Narayana et al.	2015	Epinecidin-1	Synthetically synthesized in lab	Showed bactericidal activity against drug-resistant *H. pylori* and modulated immune response in mouse-infected *H. pylori* for bacterial clearance	[35]
Xiong et al.	2017	Helix–coil conformation transitional antimicrobial polypeptides	Synthetically synthesized in lab	Displayed bactericidal activity against *H. pylori* at low pH, both in vitro and in vivo	[36]
Zhang et al.	2015	Pexiganan	Synthetically synthesized in lab	Inhibited the growth of *H. pylori* (MIC = 4 µg/mL); decreased *H. pylori* colonization in animal model	[37]
Zhang et al.	2017	Fusion PGLa-AM1	Synthetically synthesized in lab	Showed bactericidal activity against *H. pylori* in vitro and clearance of the bacteria in vivo	[38]
Makobongo et al.	2012	C_12_K-2β_12_	Synthetically synthesized in lab	Ruptured *H. pylori* surface membrane for bactericidal effect; reduced colonization of *H. pylori* in gerbil model	[39]
Rigano et al.	2012	Tomato defensin	Synthetically synthesized in lab	Showed antibacterial activity against *H. pylori* at MIC: 15 µg/mL	[40]
Iwahori et al.	1997	Magainin 2 analog	Synthetically synthesized in lab	Inhibited growth of *H. pylori* in vitro	[41]

Abbreviations: AMP: Antimicrobial peptide; MIC: Minimum inhibitory concentration.

## Data Availability

All data searched and extracted from the included studies in this systematic review are available upon request to corresponding author.
